# P-glycoprotein Inhibitor Tariquidar Potentiates Efficacy of Astragaloside IV in Experimental Autoimmune Encephalomyelitis Mice

**DOI:** 10.3390/molecules24030561

**Published:** 2019-02-03

**Authors:** Wei Zhang, Mei Liu, Liu Yang, Fei Huang, Yunyi Lan, Hongli Li, Hui Wu, Beibei Zhang, Hailian Shi, Xiaojun Wu

**Affiliations:** Shanghai Key Laboratory of Compound Chinese Medicines, the Ministry of Education (MOE) Key Laboratory for Standardization of Chinese Medicines, the State Administration of TCM (SATCM) Key Laboratory for New Resources and Quality Evaluation of Chinese Medicine, Institute of Chinese Materia Medica, Shanghai University of Traditional Chinese Medicine, Shanghai 201203, China; 13122780257@163.com (W.Z.); lmcao@hotmail.com (M.L.); yangliu996633@126.com (L.Y.); orange__fly@sina.com (F.H.); ddlanyunyi@126.com (Y.L.); lhlyunnan91@163.com (H.L.); zgykdxwuhui@foxmail.com (H.W.); 13801623470@163.com (B.Z.)

**Keywords:** astragaloside IV, P-glycoprotein, breast cancer resistance protein, blood–brain barrier, experimental autoimmune encephalomyelitis, tariquidar

## Abstract

ATP-binding cassette (ABC) transporters, such as P-glycoprotein (P-gp) and breast cancer resistance protein (BCRP), often reduce drug efficacy and are the major cause of drug resistance. Astragaloside IV (ASIV), one of the bioactive saponins isolated from *Astragalus membranaceus*, has been demonstrated to alleviate the progression of experimental autoimmune encephalomyelitis (EAE) in mice, an animal model for multiple sclerosis (MS). In the present study, we found for the first time that ASIV induced the upregulation of P-gp and BCRP in the central nervous system (CNS) microvascular endothelial cells of EAE mice. Further study disclosed that tariquidar, a P-gp inhibitor, could facilitate the penetration of ASIV into CNS. On bEnd.3 cells, a mouse brain microvascular endothelial cell line, tariquidar benefited the net uptake and transport of ASIV. Additional molecular docking experiment suggested that ASIV might be a potential substrate of P-gp. In EAE mice, tariquidar was demonstrated to enhance the efficacy of ASIV, as shown by attenuated clinical symptom and reduced incidence rate as well as mitigated inflammatory infiltration and decreased demyelination in the CNS. Collectively, our findings implicate that P-gp inhibitor can promote the therapeutic efficacy of ASIV on EAE mice, which may boost its clinical usage together with ASIV in the therapy of MS.

## 1. Introduction

Blood–brain barrier (BBB) and blood–spinal cord barrier (BSCB) are mainly composed of microvascular endothelial cells sealed by tight junctions that restrict substance influx and efflux of the central nervous system (CNS) under physiological conditions [1,2]. Although BBB and BSCB share the same principal building blocks, morphological and functional differences between them have been manifested [1]. Dysfunction of BBB and BSCB is involved in the pathological process of many neurodegenerative diseases, such as amyotrophic lateral sclerosis (ALS), multiple sclerosis (MS), and spinal cord ischemia [1,3,4,5]. The increased permeability of BBB and BSCB initially seems to facilitate therapeutic drugs to reach the CNS in neurodegenerative diseases [6,7]. However, the presence of ATP-binding cassette (ABC) transporters within BBB and BSCB throw a big challenge for the penetration of CNS drugs [8,9,10,11]. ABC transporters, the members of a transport system superfamily, include importers and exporters that consist of transmembrane proteins and membrane-associated ATPases [12]. The ATPase subunits energize the translocation of various substrates, either uptake or export, across membranes by hydrolyzing adenosine triphosphate (ATP) [13]. ABC exporters, including multidrug resistance protein (MDR), multidrug resistance associated protein (MRP), and breast cancer resistance protein (BCRP), when highly expressed, induce resistance to multiple drugs, such as antibiotics [14], antidepressants [15], anti-Parkinson’s disease (PD) candidate drug [16], and anticancer agents [17,18]. Therefore, inhibition of ABC exporters may contribute to the alleviation of drug resistance and increase drug efficacy in the CNS as well.

MS is one of the chronic neurodegenerative diseases characterized by demyelination in the CNS [19]. Experimental autoimmune encephalomyelitis (EAE) induced by myelin oligodendrocyte glycoprotein (MOG) or proteolipid protein (PLP) in mice is the most widely used animal model for the study of MS, which mimics a chronic progression or relapse–remission course of the disease [20,21,22]. ABC transporters have been implicated as being involved in pathogenesis and treatment response of MS [23]. For instance, they may modulate inflammatory processes of MS, resulting in severe neurological deficits [8]. P-gp expression has also been reported to be decreased in both MS and EAE lesions, which coincides with perivascular infiltration of lymphocyts [24]. However, so far, the exact function of ABC transporters, especially the exporters, in BBB or BSCB of MS has not been clearly elucidated.

Astragaloside IV (ASIV) is one of the active components in *Astragalus membranceus*, a well-known traditional Chinese medicine recorded in Chinese Pharmacopoeia [25]. It has been demonstrated to exert multiple pharmacological functions, including anti-obesity [26], immunoregulation [27], anti-atherosclerosis [28], antihypertension [29], antidiabetes, antioxidative stress [30,31], proneurogenesis [32], tissue protection against heart, kidney, and neuron injury [32,33,34,35], antitumor [36], anti-inflammation [37], and so on. Our previous studies showed that ASIV could alleviate the aggravation of EAE [27,38]; however, whether it induces drug resistance in EAE has not yet been reported. In the present study, the effect of ASIV on the expression of ABC exporters in the microvascular endothelial cells of BBB and BSCB was firstly investigated in EAE mice. Consequently, the P-gp inhibitor tariquidar was employed to investigate whether it could increase the penetration of ASIV into CNS and improve the therapeutic efficacy of ASIV in EAE mice. The effect of tariquidar was also evaluated on bEnd.3, a mouse brain microvascular endothelial cell line. The findings may benefit the clinical usage of ASIV together with P-gp inhibitor in the therapy of MS.

## 2. Results

### 2.1. ASIV Changed the Expression Pattern of P-gp and BCRP in BSCB but Not BBB of EAE Mice

**Figure 1:**
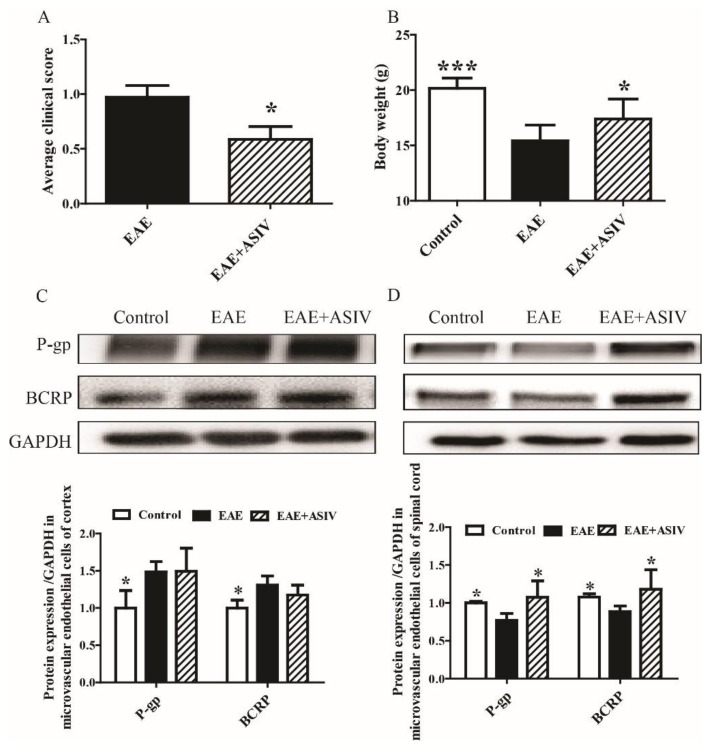
Effect of astragaloside IV (ASIV) on the expression of ATP-binding cassette (ABC) transporters in experimental autoimmune encephalomyelitis (EAE) mice. (**A**) Clinical scores of EAE mice; (**B**) body weight loss of EAE mice; (**C**) protein expression of P-glycoprotein (P-gp) and breast cancer resistance protein (BCRP) in microvascular endothelial cells isolated from cortex of EAE mouse (*n* = 5); (**D**) protein expression of P-gp and BCRP in microvascular endothelial cells isolated from spinal cord of EAE mouse (*n* = 5). Values are expressed as mean ± SD. Data were analyzed by one-way analysis of variance with Dunnett’s multiple comparison test or unpaired *t*-test. * *p* < 0.05, *** *p* < 0.001 vs. EAE group.

In agreement with previous reports, after induction for three weeks, significantly changed neurobehaviors, such as mental retardation, limb weakness, and weight loss, were observed in EAE mice. ASIV administration improved the neurobehavioral symptoms of EAE mice, as shown by decreased average clinical score from day 8 to day 18 postimmunization (Figure 1A, *p* < 0.05). Compared with the control mice, the body weight of EAE mice was decreased remarkably on day 18 postimmunization (Figure 1B, *p* < 0.001), which could be reversed by ASIV administration (Figure 1B, *p* < 0.05). EAE induction resulted in the alteration of ABC exporters in microvascular endothelial cells of BBB and BSCB. As shown in Figure 1C, the levels of P-gp and BCRP were increased markedly in BBB microvascular endothelial cells isolated from EAE mice (*p* < 0.05). However, in BSCB microvascular endothelial cells of EAE mice, the expression of P-gp and BCRP were decreased significantly (*p* < 0.05). ASIV administration did not decrease the expression of P-gp and BCRP in BBB microvascular endothelial cells of EAE mice. Nevertheless, ASIV induced the expression of P-gp and BCRP in BSCB microvascular endothelial cells of EAE mice (Figure 1D, *p* < 0.05). 

### 2.2. Tariquidar Facilitated the Penetration of ASIV into CNS of EAE Mice

**Figure 2:**
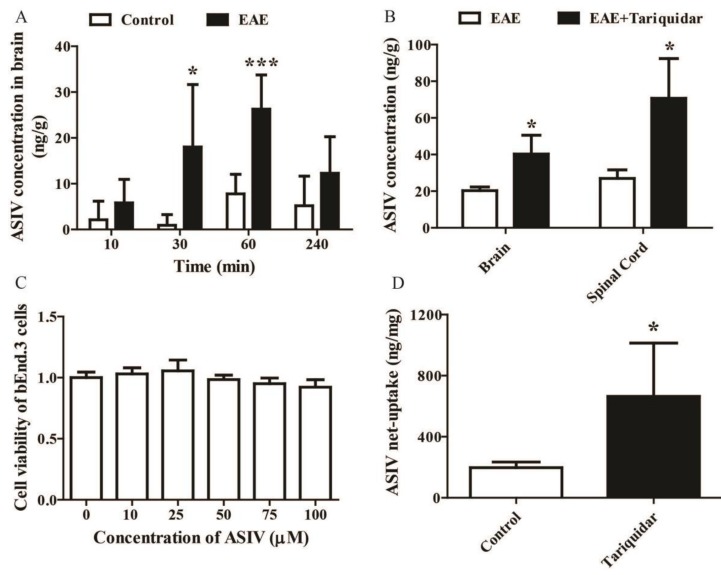
Tariquidar enhances the net uptake of ASIV into brain and spinal cord of EAE mice. (**A**) Time course comparison of the penetration of ASIV into brain parenchayma of control and EAE mice after single administration (*n* = 6); (**B**) effect of tariquidar on the penetration of ASIV into brain and spinal cord of EAE mice (*n* = 10); (**C**) effect of ASIV on cell viability of bEnd.3 cells; (**D**) effect of tariquidar on the net uptake of ASIV in bEnd.3 cells. Values are expressed as mean ± SD. Data were analyzed by one-way analysis of variance with Dunnett’s multiple comparison test or unpaired *t*-test. * *p* < 0.05, *** *p* < 0.001 vs. control group.

In order to evaluate whether EAE induction could increase the penetration of ASIV into CNS, the concentrations of ASIV in brain parenchyma of EAE mice after intraperitoneal drug administration for different time points were detected by LC-MS/MS. As shown in Figure 2A, the concentration of ASIV in brain parenchyma of EAE mice was increased gradually and reached its peak (26.28 ng/g) within 60 min, then decreased slowly at 240 min after injection. Interestingly, the concentration of ASIV in brain parenchyma of the control mice also attained its peak (7.78 ng/g) after drug administration for 60 min. Therefore, the time point, namely, 60 min after drug administration, was chosen for the following experiments. As shown in Figure 2B, when tariquidar, the P-gp inhibitor, was used, the concentrations of ASIV penetrated into the brain and spinal cord of EAE mice were increased more than 1-fold (Figure 2B, *p* < 0.05).

To investigate whether tariquidar could facilitate the net uptake of ASIV into brain microvascular endothelial cells, the concentrations of ASIV in bEnd.3 cells pretreated with tariquidar were examined. As displayed in Figure 2C, ASIV ranging from 10 μM to 100 μM did not affect the cell viability of bEnd.3 cells. The basal net uptake of ASIV by bEnd.3 cells was about 197 ng/mg after treatment with 50 μM ASIV for 1 h (Figure 2D). However, after being pretreated with tariquidar, the net uptake of ASIV by bEnd.3 cells was increased to 665 ng/mg, which was significantly different from the control (Figure 2D, *p* < 0.05). 

**Figure 3:**
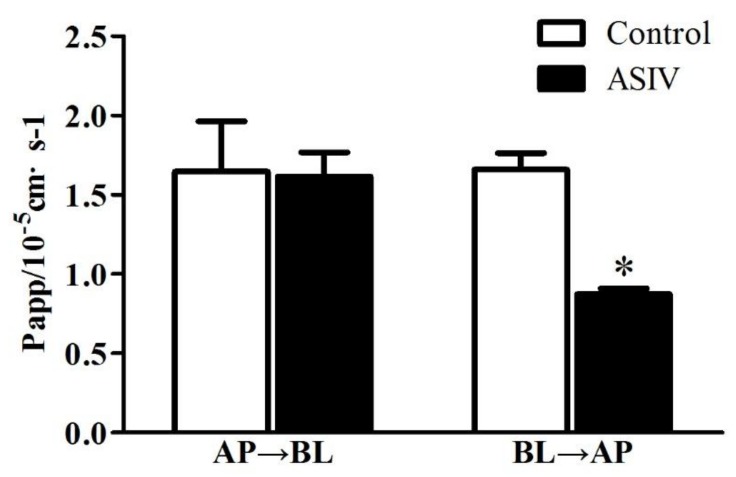
Effect of tariquidar on the transportation of ASIV across bEnd.3 cells. Values are expressed as mean ± S.D. (*n* = 3). Data were analyzed by unpaired *t*-test. * *p* < 0.05 vs. control group. AP→BL: permeability of ASIV from apical side to basal side. BL→AP: apparent permeability of ASIV from basal side to apical side.

To identify whether P-gp inhibitor could also affect the transportation of ASIV through microvessel endothelial cells, the effect of tariquidar on the transportation of ASIV through bEnd.3 cells was examined. As revealed in Figure 3, the addition of tariquidar did not change the apparent permeability of ASIV from the apical (AP) side to the basal (BL) side. However, it significantly decreased the apparent permeability of ASIV from the BL side to the AP side (*p* < 0.05). All of these results implicate that P-gp inhibitor can decrease the efflux of ASIV from CNS and thus increase the penetration or absorption of ASIV in the CNS.

### 2.3. ASIV Was a Potential Substrate of P-gp

**Figure 4:**
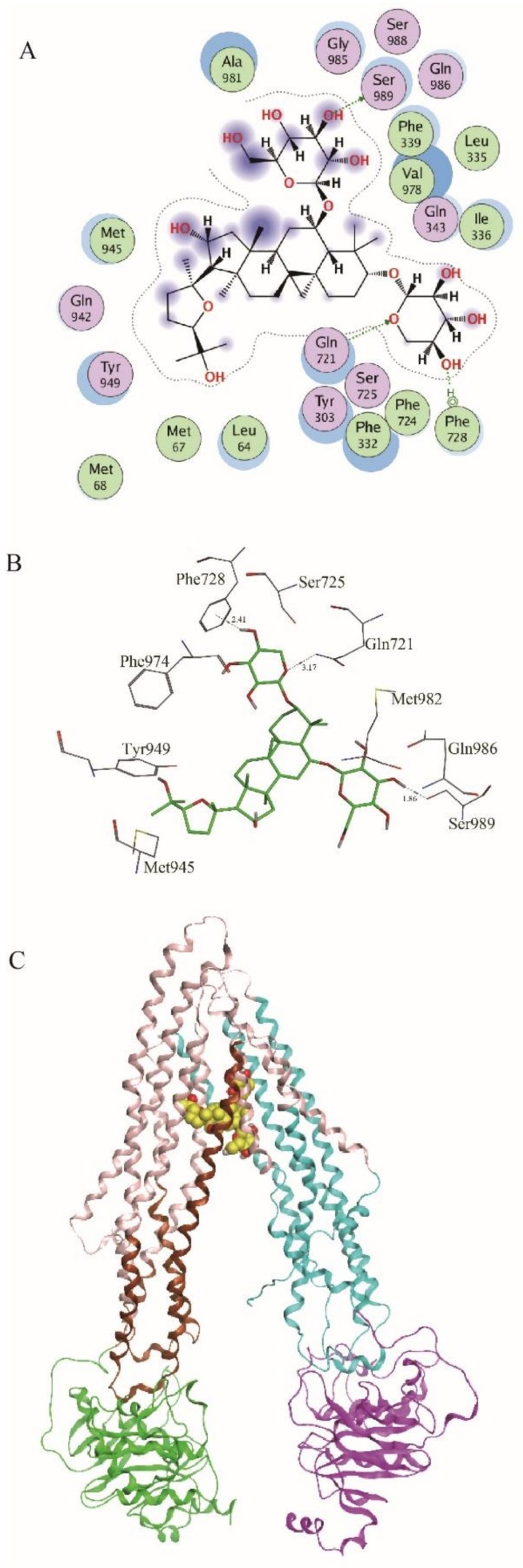
Molecular docking experiment. (**A**) 2D interaction map of the interactions between ASIV and P-gp; (**B**) the binding mode of ASIV and P-gp structure; (**C**) the whole view of the binding mode of ASIV and P-gp structure.

Molecular docking was performed to investigate if ASIV could bind to P-gp using MOE-Dock. Site Finder was used to identify the potential binding sites. The most likely drug-bound pocket contains the critical residues Met68, Phe332, Ile336, Phe339, Leu335, Gln721, Phe728, Phe724, Phe833, and Ser989. The final predicted binding poses were ranked by GBVI/WSA dG score. In total, 20 docking poses were selected for further analysis. The poses ranked at first, second, fourth, fifth, tenth, eleventh, and twentieth shared similar binding mode as the P-gp protein structure. The 2D interaction map of the best ranking pose showed that there were two hydrogen bonds formed between ASIV and Gln721 and Ser989 (Figure 4A); the distances of these two hydrogen bonds were 2.31Å and 1.86Å, respectively (Figure 4B). ASIV in the largest site that bound to the C-terminal half of the TMD (Figure 4C) was in close proximity to TMs 1, 5, 6, 7, 8, 9, 11, and 12 and surrounded by the upper two polar residues (Gln721 and Ser989). In addition, an arene–H (H–π) interaction was formed between ASIV and Phe728 of P-gp, which plays an important role in stabling the binding of ASIV with P-gp (Figure 4A). The distance of this H–π interaction was 2.41Å (Figure 4B). In the binding site, ASIV was surrounded by the polar residues Gln343, Gln721, Ser725, Gln942, Tyr949, Gly985, and Ser989. The results suggested that ASIV might be a substrate of P-gp.

### 2.4. Tariquidar Potentiated the Efficacy of ASIV on EAE Mice

**Figure 5:**
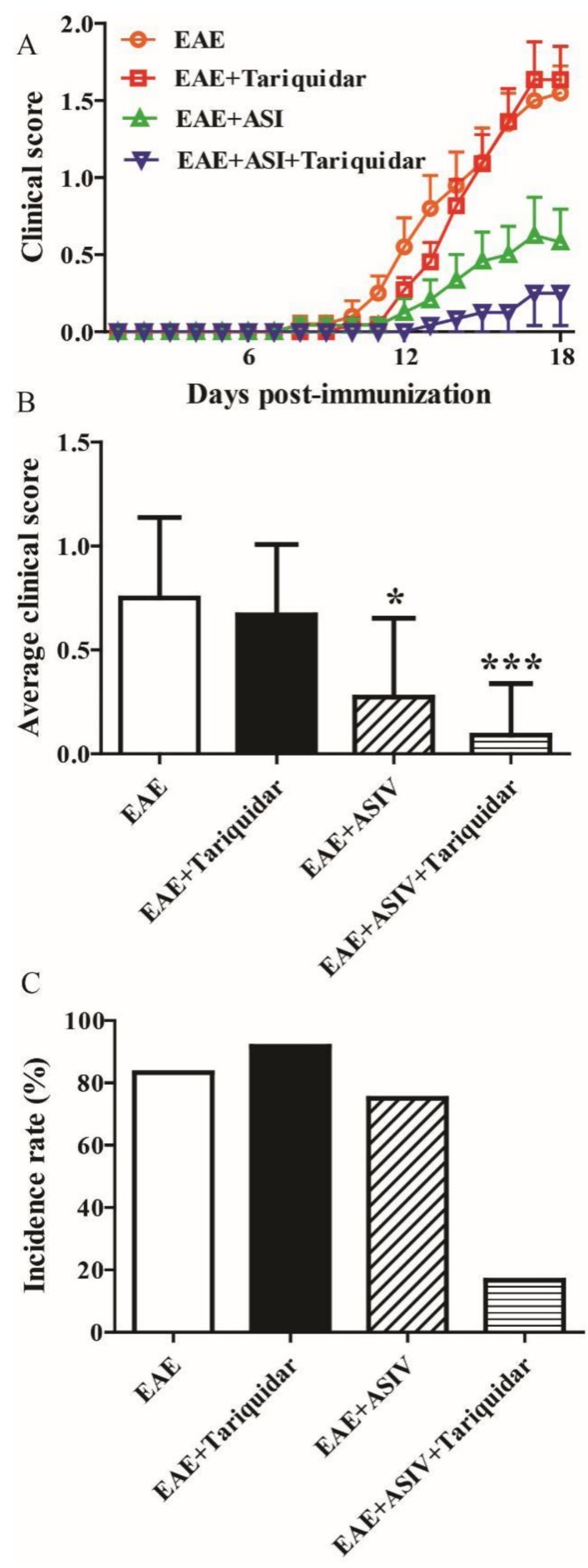
P-gp inhibitor enhances the alleviative effect of ASIV on EAE mice. (**A**) Effects of ASIV combined with tariquiar on the neurobehavior of EAE mice (*n* = 12); (**B**) effect of ASIV combined with tariquiar on the average clinical score of EAE mice (*n* = 12); (**C**) effect of ASIV combined with tariquiar on the EAE incidence rate (*n* = 12). Values are expressed as mean ± SD. Data were analyzed by one-way analysis of variance with Dunnett’s multiple comparison test. * *p* < 0.05, *** *p* < 0.001 vs. EAE group.

As shown in Figure 5A, tariquidar alone had no significant effect on the clinical score of EAE mice. ASIV significantly prevented the exacerbation of EAE as aforementioned. Meanwhile, coadministration of tariquidar seemed to potentiate the preventive effect of ASIV on EAE progression. Accordingly, the average clinical score from day 8 to day 18 postimmunization of ASIV and tariquidar cotreated EAE mice appeared to be lower when compared with the ASIV-treated ones (Figure 5B). Moreover, the incidence rate of ASIV and tariquidar cotreated mice dropped to 16.7% compared with mice treated with ASIV alone (75%) (Figure 5C). 

**Figure 6:**
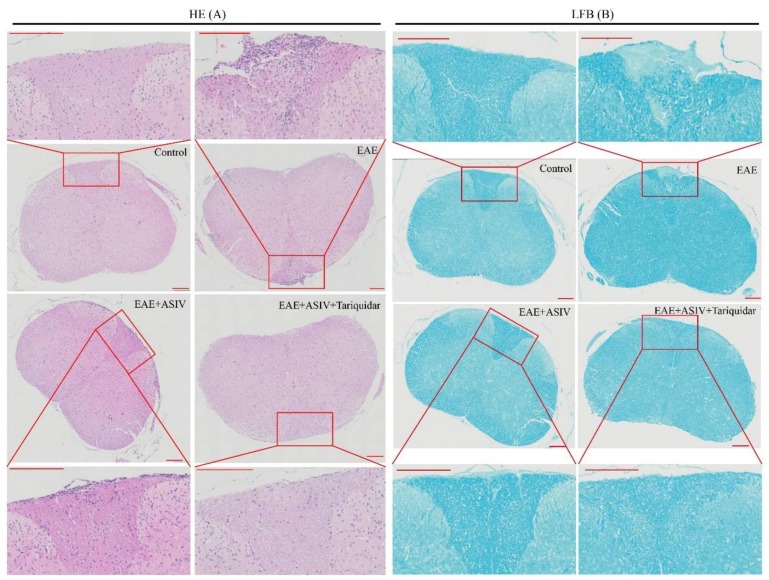
Tariquidar enhances the alleviative effect of ASIV on the inflammatory cell infiltration and demyelination of spinal cord in EAE mice. (**A**) Effect of coadministration of ASIV and tariquiar on the inflammatory cell infiltration. The spinal cord sections were stained by hematoxylin and eosin (H&E) staining method. (**B**) effect of coadministration of ASIV and tariquiar on the demyelination. The spinal cord sections were stained by luxol fast blue (LFB) staining method. Scale bar: 200 μm.

In terms of inflammatory infiltration, as shown in Figure 6A, ASIV alone effectively decreased the number of infiltrated mononuclear cells. However, coadministration with tariquidar seemed to enhance the inhibitory effect of ASIV. Accordingly, luxol fast blue (LFB) staining demonstrated that ASIV alone attenuated the demyelination process. Coadministration with tariquidar enhanced the alleviative effect of ASIV on demyelination of the spinal cord in EAE mice (Figure 6B). 

## 3. Discussion

BBB and BSCB are pivotal regulators of CNS homeostasis, which forms a crucial interface regulating the molecular flux between blood and brain or spinal cord [1,2]. ABC transporters at the luminal membrane [39] are in charge of the efflux of molecules from the microvasular endothelium of BBB and BSCB and have been indicated to protect CNS against neurodegenerative diseases under pathological conditions, such as ALS, PD, and AD [40,41,42,43]. As aforementioned, vascular P-gp expression is decreased in lesions of MS patients and EAE rats, and its disappearance is relevant to the perivascular infiltration of lymphocytes [24]. In addition, the loss of P-gp slightly aggravates EAE progression in mice as it decreases the secretion of proinflammatory cytokines, such as TNFα and IFNγ [44]. In the present study, different expression patterns of P-gp and BCRP were found between BBB and BSCB endothelial cells of EAE mice. In brain microvascular endothelial cells of EAE mice, the ABC exporters were revealed to be upregulated. In contrast, in the spinal cord microvascular endothelial cells of EAE mice, the expression of P-gp and BCRP were decreased. As suggested by Bartanusz et al. [1], specific differences exist in the ultrastructure of BBB and BSCB endothelial cells, which might reflect substantial physiological differences between the two barrier systems. The different expression pattern of ABC exporters within BBB and BSCB endothelical cells of EAE mice might partially disclose the different pathophysiological roles of these two barriers.

Drug resistance resulting from ABC transporters is a big universal challenge for the treatment of neurodegenerative diseases [7]. Different ABC exporters may exert completely different functions. For instance, P-gp participates in the rapid removal of ingested toxic lipophilic metabolites, including amphipathic cationic drugs [39,45]. In comparison, several MRPs including BCRP expressed in the microvessels of brain mediate mainly the efflux of anionic compounds. These exporters work together, accounting for the reduced penetration but increased efflux of many drugs from CNS [46]. In the present study, ASIV was demonstrated to pass through BBB and penetrate into the brain parenchyma of EAE mice. ASIV has been reported to induce the expression of P-gp and BCRP in liver tissues [47]. To further investigate if it induces possible drug resistance in BBB and BSCB, the expression of P-gp and BCRP after ASIV treatment was examined. Compared to the EAE group, ASIV did not change the expression of P-gp and BCRP in brain microvascular endothelial cells but increased that in spinal cord microvascular endothelial cells, suggesting ASIV at least partially induces drug resistance in EAE mice by increasing ABC exporters. Thereafter, the P-gp inhibitor tariquidar was employed. The results showed that tariquidar time-dependently increased the accumulation of ASIV in brain parenchyma. Accordingly, coadministration of tariquidar enhanced the efficacy of ASIV in EAE mice, as evidenced by reduced clinical score, EAE incidence, infiltration of inflammatory cells, and demyelination. In agreement with the in vivo experimental results, tariquidar could enhance the net uptake of ASIV by bEnd.3 cells and reduce the transportation of ASIV from the BL to the AP side. Furthermore, the molecular docking study suggested that ASIV was a potential substrate transported by P-gp. All of these results implicate that ABC exporters actively participate in the cellular exportation of ASIV in BBB and BSCB microvascular endothelium. 

**Figure 7:**
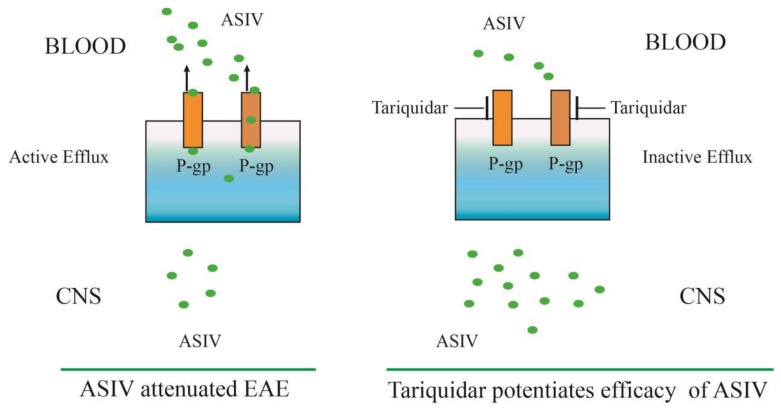
Schematic illustration of the effect and mechanism of tariquidar, a P-gp inhibitor, in potentiating the efficacy of ASIV on EAE. P-gp was significantly increased in microvascular endothelial cells from the cortex in EAE mice, which might compromise the efficacy of ASIV on EAE. Coadministration of tariquidar potentiated the neuroprotective effect of ASIV in EAE mice by reducing the efflux of the penetrated ASIV in the CNS.

In conclusion, our results provide insight into the role of ASIV in upregulating P-gp and BCRP expression in microvascular endothelial cells from the spinal cord of EAE mice, which was found to attenuate EAE in our previous studies. However, the inhibitor of P-gp (tariquidar) could significantly increase the accumulation of ASIV in the CNS in vitro and in vivo and improve the therapeutic efficacy of ASIV on EAE. Figure 7 summarizes the effect and mechanism of tariquidar in potentiating the efficacy of ASIV on EAE. Taken together, our results demonstrate that inhibition of BBB and BSCB microvascular endothelial P-gp can enhance the efficacy of ASIV on EAE. The novel findings may provide an alternative therapeutic regimen for MS patients.

## 4. Materials and Methods

### 4.1. Reagents

ASIV (Cat. No. 160509) was purchased from Shanghai Lightgoal Industry Co. LTD with a purity of 98.9%. Tariquidar (Cat. No. S8028) was obtained from Selleck Chemicals (Houston, TX, USA). Fetal bovine serum (FBS) (Cat. No. 8172881) and 0.25% trypsin (Cat. No. 25200072) were purchased from Life Technology (Carlsbad, CA, USA). P-gp antibody (Cat. No. ab170904) was purchased from Abcam (Cambridge, MA, USA). BCRP antibody (Cat. No. GTX60447) was purchased from GeneTex (Irvine, CA, USA). Antibody against glyceraldehyde 3-phosphate dehydrogenase (GAPDH) (Cat. No. 5174) was provided by Cell Signaling Technology (Danvers, MA, USA). Goat anti-rabbit IgG secondary antibody (Cat. No. 111-035-003) was obtained from Jackson Immuno Research (West Grove, PA, USA). CellLytic^TM^ MT mammalian tissue lysis reagent (Cat. No. C3228) was purchased from Sigma (Saint Louis, MO, USA). Protease inhibitor cocktails (Cat. No. 04693116001) and phosphates inhibitor cocktails (Cat No. 04906837001) were purchased from Roche (Mannheim, Germany). Cell Counting Kit-8 (CCK-8) (Cat. No. CK04) was purchased from Dojindo Laboratories (Kumamoto, Japan). 

### 4.2. Animals

Female C57BL/6 mice (18-22 g, 6 weeks old) were provided by the Laboratory Animal Center of Shanghai University of Traditional Chinese Medicine (SHUTCM, shanghai). The animals were cultivated under a 12 h light/dark cycle at room temperature (22 ± 1 °C) with access to food and water ad libitum. All animal experiment protocols were conducted in compliance with the Institutional Animal Care guidelines approved by the Experimental Animal Ethical Committee (Shanghai, China) at SHUTCM (approval no. PZSHUTCM18110101, 1 January 2016). All animal experiments were also performed in compliance with the National Institutes of Health Guide for the Care and Use of Laboratory Animals (NIH Publications No. 8023, revised 1978).

### 4.3. EAE Induction and Treatment

EAE was induced in mice by subcutaneous immunization with myelin oligodendrocyte glycoprotein 35–55 (MOG_35–55_) as described previously [27,38]. The control mice were induced with the same reagents except MOG_35–55_. Clinical behavior of mice was scored daily in compliance with the criteria used by Peiris et al. [48]. ASIV treatment (20 mg/kg) was given intraperitoneally (i.p.) daily from the day before MOG_35–55_ immunization and continued for 18 days. Tariquidar (13 mg/kg) was given intraperitoneally (i.p.) 30 min before ASIV administration one time per two days for 18 days.

### 4.4. Histopathology

After anesthetization with excessive 20% urethane, the mice were perfused intracardially with phosphate buffered saline (PBS) followed by 4% paraformaldehyde. The dissected spinal cords were fixed in 4% paraformaldehyde, dehydrated in 15% and 30% sucrose PBS solution, and cut into 20 μm coronal sections. Thereafter, the sections were subjected to haematoxylin and eosin (H&E) staining and luxol fast blue (LFB) staining as described previously [27,38]. The images were taken by Olympus VS120 Virtual Slide Scanner.

### 4.5. Microvessel Isolation

Microvessels in cerebral cortices and spinal cords of mice were prepared according to the method described previously [49]. In brief, brain cortices were dissected and homogenized in capillary buffer (10 mM HEPES, 141 mM NaCl, 4 mM KCl, 2.8 mM CaCl_2_, 1 mM NaH_2_PO_4_, 1 mM MgSO_4_, 10 mM d-glucose, pH 7.4) with Dounce glass homogenizers. The homogenate was mixed thoroughly with 26% dextran and centrifuged at 9000× *g* for 30 min at 4 °C. The pellet with enriched endothelial fraction was washed with PBS and then stored at −80 °C for further analysis.

### 4.6. LC-MS/MS Analysis

Normal mice and EAE mice were i.p. administered with ASIV (60 mg/kg for normal mice, 40 mg/kg for EAE mice) pretreated with or without tariquidar (13 mg/kg) for 20 min. One hour later, the mice were sacrificed with excessive pentobarbital sodium. The whole brain was dissected out and separated into two equal parts. Each half of the brain was homogenized in a disposable glass tube after addition of 800 μL methanol and 200 μL distilled water. The homogenate was vortex-mixed for 1 min and then centrifuged at 18,000× *g* for 10 min at 4 °C. The supernatant was transferred and evaporated to dryness under nitrogen stream. The dry residue was reconstituted in 100 μL of initial mobile phase (0.1% formic acid in water/acetonitrile, 70:30, *v*/*v*), and its aliquot (10 μL) was injected into the LC-MS system for analysis.

LC-MS/MS was run on an Agilent 6410 triple stage quadrupole mass spectrometer equipped with an ESI ion source and an Agilent 1290 HPLC system with autosampler (Agilent Technologies, Santa Clara, CA, USA). The analytes were separated on a BEH C_18_ column (2.1 mm × 100 mm, 1.7 μm, Waters, Milford, USA) used at 30 °C. The mobile phase consisting of 0.1% formic acid in water (Solvent A) and acetonitrile (Solvent B) was used with a gradient elution 0–4 min, 30% B; 5 min, 60% B; 6–8 min, 90% B, 11 min, 30% B at a flow rate of 0.2 mL/min. ESI-MS/MS conditions were set as follows: gas temperature 325 °C, gas flow 10 L/min, capillary 4000 V, and nebulizer pressure 35 psi. 

### 4.7. Western Blot Analysis

Cerebral and spinal cord capillary were isolated according to the method described by Yu et al. [49] and lysed in CellLytic^TM^ MT mammalian tissue lysis reagent (Sigma) containing protease inhibitor cocktail (Roche) and phosphates inhibitor cocktail 2 (Roche). Afterward, the lysis was centrifuged at 12,000 rpm and 4 °C for 15 min. Protein concentration of the supernatants was quantified by bicinchoninic acid assay (BCA) method. Equal amounts of protein lysates (20 μg) were subjected to a 10% SDS-PAGE gel electrophoresis. Then, proteins were transferred onto Fluoro Trans polyvinylidene difluoride (PVDF) membranes (Pall, 20685) by an electrophoretic transfer system (Bio-Rad, Hercules, CA, USA). The membranes were blocked with 5% milk in PBS with tween-20 (PBST) for 1 h and then incubated with primary antibodies at 4 °C overnight. After being thoroughly washed three times with PBS containing 0.1% Tween 80, the membranes were further incubated with horseradish peroxidase (HRP)-conjugated secondary antibodies and detected with enhanced chemiluminescence (ECL) system (Amersham Biosciences, Pitscataway, NJ, USA). All the primary antibodies were purchased from C.S.T (Beverly, MA, USA).

### 4.8. Cell Culture

bEnd.3 cells obtained from ATCC were cultured in RPMI-1640 medium containing 25 mM 4-(2-hydroxyethyl)-1-piperazineethanesulfonic acid (HEPES) (WisentInc, Nanjing, China), supplemented with 10% FBS (Gibco, Waltham, MA, USA) and 1% penicillin/streptomycin. The cells were cultured at 37 °C in a humidified incubator with 5% CO_2_.

### 4.9. CCK-8 Assay

One hundred microliters of bEnd.3 cells were seeded at a density of 1 × 10^5^ cells/mL in 96-well plate (Corning). After being cultured overnight, the cells were treated with ASIV (0, 25, 50, 75 and 100 μM) for 24 h. Then, 10 μL of CCK-8 solution was added to each well and incubated at 37 °C for another 1 h. The absorbance of the solutions was detected at 450 nm by a microplate reader (Thermo Scientific Varioskan Flash, Waltham, MA, USA). The cell viability rate was calculated as follows: (absorbance of drug-treated sample / absorbance of control sample) × 100.

### 4.10. ASIV Net Uptake Assay 

bEnd.3 cells were seeded at a density of 1 × 10^5^ cells/mL in 6-well plates (Corning), and cultured for 4 days with medium refreshment per 2 days. Then, the medium was removed, and the cells were washed with Hanks’ balanced salt solution (HBSS) three times. Cells were pretreated with or without tariquidar (5 μM) in HBSS for 30 min, followed by washing with HBSS once. Then, the cells were treated with or without ASIV (50 μM) in HBSS for 1 h. The cells were washed with HBSS three times and then lysed in deionized water (0.4 mL/well) by repeated freezing and thawing three times at −80 °C followed by sonication for 15 min. The suspension (0.2 mL/well) was pipetted and mixed with anhydrous methanol (0.8 mL) and centrifuged at 20,000 rpm for 10 min at 4 °C. The supernatant was purged with nitrogen and then resuspended with 100 μL solution containing methanol: 0.1% formic acid in water (3:7), vortexed and homogenized at 20,000 rpm for 10 min at 4 °C. The ASIV content in the supernatant was detected by LC-MS/MS method. Net uptake of ASIV by bEnd.3 cells was shown as ng/mg protein. 

### 4.11. ASIV Transmembrane Absorption

The cells were seeded at a density of 1 × 10^5^ cells/mL in 24-well transwell inserts (pore size 0.4 μm, Corning). After being cultured for 10 days, the cells were washed with HBSS three times, and the transendothelial electrical resistance (TEER) was measured with an electrical resistance system (Millicell ERS-2 epithelial volt-ohm-meter, Millipore, Burlington, MA, USA). Once the TEER was above 50 Ω·cm^2^, the cells were treated with ASIV (50 μM) and/or tariquidar (5 μM) added in the apical (AP) or the basal (BL) side. Transmembrane absorption of ASIV was detected at 30, 60, 90, and 120 min after ASIV treatment. TEER was measured at 120 min after ASIV treatment. Papp was calculated as following: Papp = (dQ/dt)/(A × C_0_), where dQ/dt represents drug delivery per unit time (μg·s^−1^), A is the membrane area (0.3 cm^2^ in the present study), and C_0_ is the starting concentration of drug (μg·mL^−1^).

### 4.12. Molecular Docking Experiment

The drug-bound P-gp structure (PDB code: 3G60, Resolution: 4.4Å) was obtained from Protein Data Bank (PDB) [50]. The cocrystalized structure was prepared using MOE 2016.08 [51] for correcting structural issues (such as break bond, miss loop, etc.), adding hydrogen, and calculating partial charge. The 2D structure of the ASIV was downloaded from the PubChem database [52] with sd file format and converted to 3D in MOE through energy minimization. MOE-Dock was used for docking simulation of the ASIV and predicting the binding affinity with the P-gp protein structure. The original drug-binding pocket was chosen as the active site for docking. Site Finder in MOE was also used to identify the potential binding pockets and analyze the conserved pocket residues. Classical triangle matching was chosen as placement method, and the number of placement poses was set to 100. The output docking poses were evaluated by the London dG score. Then, the rigid receptor method was employed in the refinement step. The number of the final output docking poses was set to 20, followed by minimizing using Amber12: EHT force field in MOE. The GBVI/WSA dG score was used to evaluate the binding of the ASIV with the P-gp. The binding mode was analyzed in MOE after the refinement minimization.

### 4.13. Statistical Analysis

All data in the graphs were presented as mean ± standard deviation (SD). Differences among groups were analyzed by one-way ANOVA with Dunnett’s multiple comparison test using GraphPad Prism 5 (GraphPad Software, La Jolla, CA, USA). Comparisons between two groups were conducted using unpaired student *t*-test. *p* < 0.05 was considered statistically significant.
